# Shear-Thinning and Temperature-Dependent Viscosity Relationships of Contemporary Ocular Lubricants

**DOI:** 10.1167/tvst.11.3.1

**Published:** 2022-03-02

**Authors:** Wasim Kapadia, Ning Qin, Pei Zhao, Chau-Minh Phan, Lacey Haines, Lyndon Jones, Carolyn L. Ren

**Affiliations:** 1 Department of Mechanical and Mechatronics Engineering, University of Waterloo, Waterloo, ON, Canada; 2 Centre for Ocular Research & Education (CORE), School of Optometry & Vision Science, University of Waterloo, Waterloo, ON, Canada; 3 Centre for Eye and Vision Research (CEVR), Hong Kong; 4 School of Energy and Power Engineering, Shandong University, Jinan, Shandong, China

**Keywords:** artificial tears, ocular lubricants, viscosity, shear rates, temperature-dependence

## Abstract

**Purpose:**

To evaluate the shear viscosity of contemporary, commercially available ocular lubricants at various shear rates and temperatures and to derive relevant mathematical viscosity models that are impactful for prescribing and developing eye drops to treat dry eye disease.

**Methods:**

The shear viscosity of 12 ocular lubricants was measured using a rheometer and a temperature-controlled bath at clinically relevant temperatures at which users may experience exposure to the drops (out of the refrigerator [4.3°C]; room temperature [24.6°C]; ocular surface temperature [34.5°C]). Three replicates for each sample at each temperature were obtained using a standard volume (0.5 mL) of each sample. The viscosity of each ocular lubricant was measured over the full range of shear rates allowed by the rheometer.

**Results:**

The shear viscosity of the same ocular lubricant varied significantly among the three temperatures. In general, a higher temperature resulted in smaller viscosities than a lower temperature (an average of −48% relative change from 4.3°C to 24.6°C and −21% from 24.6°C to 34.5°C). At a constant temperature, the viscosity of an ocular lubricant over the studied shear rates can be well approximated by a power-law model.

**Conclusions:**

Rheological analysis revealed that the ocular lubricants exhibited shear-thinning behavior at the measured temperatures. Differences in the ocular lubricants’ formulations and measured temperatures resulted in different viscosities.

**Translational Relevance:**

When prescribing eye drops, eye care professionals can select the optimal one for their patients by considering a variety of factors, including its rheological property at physiologically relevant shear rates and temperatures, which can improve residence time on the ocular surface, while ensuring appropriate comfort and vision. However, care must be taken when using the derived mathematical models in this study because the in vivo shear behavior of the ocular lubricants has not been examined and might show deviations from those reported when placed on the ocular surface.

## Introduction

Dry eye disease (DED), which affects an estimated 25 to 30 million individuals globally,^1^ is a multifactorial disease characterized by ocular discomfort, photophobia, visual disturbance, irritation, and burning.^2^^,^^3^ It is generally believed to be the result of a combination of excessive tear evaporation and insufficient tear production.^4^^–^^6^ A poor quality tear film can lead to damage of the underlying epithelial cells,^7^ causing further tear film instability and inflammation,^8^ exacerbating the disease in a persisting cycle.^3^^,^^7^^,^^9^

One of the first-line treatments for DED is the use of ocular lubricants (also commonly referred to as artificial tears or eye drops).^3^^,^^10^ Typically, artificial tears are composed of electrolytes, surfactants, lubricants, and preservatives. Once applied, the lubricants spread evenly over the ocular surface to provide relief of inflammation and minimize friction between the eyelid and surface of the eye during blinking and to stabilize the tear film.^4^^,^^11^ Manufacturers typically vary the physical properties of artificial tear formulations by using different lubricants, thereby changing their mechanism of action.^4^ Users generally tend to choose an artificial tear formulation based on a qualitative assessment of its comfort, blur potential once instilled onto the eye, and reduction of dry eye symptoms.^12^^–^^18^

Artificial tears only provide short-term relief on administration because they are rapidly removed from the ocular surface through tear drainage, nonproductive absorption, and blinking.^18^^–^^23^ Furthermore, there is a physical limit to the volume of tears that the cul-de-sac can hold, which is typically 30 µL, and any overflow amount is drained away.^24^ To increase the residence time and bioavailability of artificial tears, one strategy is to increase the viscosity of the formulations using high molecular weight polymers.^24^

Although increasing viscosity improves residence time, it also can create several problems such as discomfort and blurring. In theory, the ideal formulation should be viscous at low shear rates, such as when the eyes are opened, to prevent removal of the lubricants from the ocular surface.^25^ However, during high-shear conditions, such as blinking, the viscosity should be low to minimize friction, discomfort, and ocular drainage.^26^ Therefore non-Newtonian artificial tears with a shear-thinning behavior (i.e., viscosity reduces with increased shear rates) are desirable.

Temperature has a significant effect on the viscosity of artificial tears.^7^ In general, as the temperature of a non-Newtonian fluid is increased, its viscosity decreases.^27^ For instance, the viscosity of an eye drop will be lower at ocular surface temperature than if it was stored in a room-temperature bottle.^28^ Additionally, some clinicians advise patients to store their eye drops in the refrigerator (at approximately 4°C) for more pronounced relief of dry eye symptoms on insertion, because of its cool sensation. It is expected that chilled artificial tear formulations will have an increased viscosity and ultimately a longer residence time on instillation. However, this may result in increased blurring of vision.

Viscosity data of commercially available eye drops at various temperatures may aid eye care practitioners in their choice of a suitable ocular lubricant. However, the viscosity information for common, contemporary over-the-counter eye drops is not readily available and is often not provided by the manufacturer. A recent study reported a novel approach of identifying the effective tear extensional viscosity using acoustic-driven microfluidic approach, which, however, is based on patient tear films.^29^ In addition, to the best of our knowledge, the temperature effects on the viscosity of commercial eye drops have not been evaluated. The purpose of this study was to provide important information on these two points by evaluating the shear viscosity of commercially available eye drops at various clinically relevant temperatures and shear rates and derive relevant mathematical viscosity models that are impactful for prescribing and developing eye drops to treat DED.

## Materials and Methods

**Figure 1.**
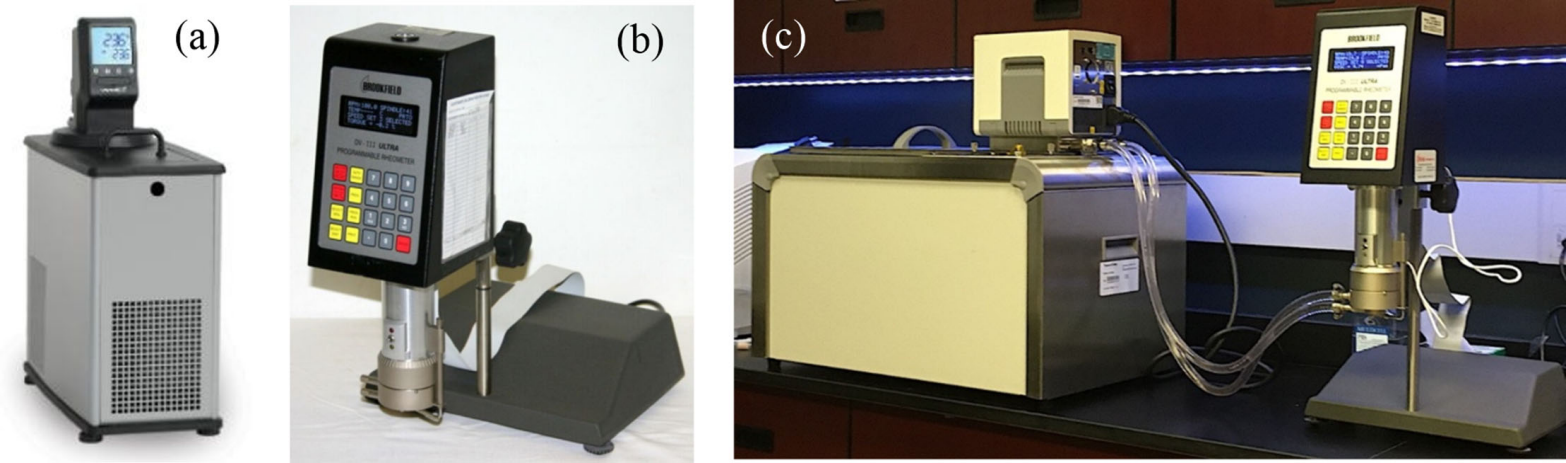
The shear viscosity measurement setup: (a) the rheometer; (b) a refrigerated bath circulator (model: mx 71 r-20; VWR International, Radnor, PA, USA); and (c) a warming bath circulator (left; model: SC150-S45; Thermo Fisher Scientific, Waltham, MA, USA) connected to the rheometer.

A total of 12 commercially available eye drops (Table 1) were evaluated in this study. The selection of these eye drops was made in such a way as to provide a wide range of viscosities from around 1 to >100 mPa · s. Shear viscosity was measured by using a cone/plate rheometer (Model: LVDV-III+; Brookfield Engineering Laboratories Inc, Middleboro, MA, USA) equipped with a standard cup and a resistance temperature detector (RTD) probe (Model: CPA-44PYZ) (Fig. 1). The spindle made a cone angle of 0.8° with the bottom plate and had a cone radius of 2.4 cm. The rheometer's built-in electronic gap setting feature was used to establish a 0.0005-inch spacing between the cup and the spindle every time the spindle was attached.

**Table 1.** Ocular Lubricants Used in Study

Name	Manufacturer	Lubricant
Systane Balance Lipid Layer Formula	Alcon Laboratories Inc.	Propylene glycol
Thealoz Duo	Théa Pharmaceuticals Limited	Trehaloze Sodium hyaluronate
Systane Complete	Alcon Laboratories Inc.	Propylene glycol Hydroxypropyl guar
Refresh Optive Advanced	Allergan Inc.	Polysorbate 80: 0.5% Carboxymethylcellulose Sodium: 0.5% Glycerine: 1%
Long Lasting Relief	Hydrasense	Sodium hyaluronate: 0.15%
Hylo Comod	Hylo Eye Care	Sodium hyaluronate: 0.1%
Bio True	Bausch + Lomb	Sodium hyaluronate: 0.24%
Blink Moisturizing Lubricant Eye drops	Johnson & Johnson	Polyethylene glycol 400 Sodium hyaluronate
Systane Ultra High Performance	Alcon Laboratories Inc.	Polyethylene glycol 400: 0.4% Propylene glycol: 0.3%
Refresh Optive Fusion	Allergan Inc.	Carboxymethylcellulose Sodium: 0.5% Glycerine: 0.9%
Systane Ultra Hydration	Alcon Laboratories Inc.	Sodium hyaluronate Hydroxypropyl guar
Refresh Optive Gel Drops	Allergan Inc.	Carboxymethylcellulose Sodium: 1% Glycerine: 0.9%

Shear viscosities were obtained at three clinically relevant temperatures at which users may experience exposure to the drops. Most eye drops are stored and applied at room temperature (∼24.6°C); however, some eye care practitioners advise their patients to refrigerate their eye drops before application, resulting in eye drops that have temperatures close to 4.3°C.

Finally, on application, eye drops will heat up to the ocular surface temperature (∼34.5°C). Understanding the shear viscosity information at these three temperatures is therefore important when prescribing eye drops for treating DED or for developing new formulations.

Using a micropipette, 0.5 mL of the sample was collected and deposited into the cup. The temperature displayed on the rheometer was recorded. For each shear viscosity measurement, the spindle rotation speed (revolutions per minute [RPM]) was manually inputted and the resulting torque (%), viscosity (mPa · s), shear stress (N/m^2^), and shear rate (s^−1^) were calculated and displayed by the rheometer and then recorded. To ensure a valid measurement, the torque reading needed to be between 10% and 100%, as per the instructions from the rheometer manufacturer. Therefore, every time a new type of eye drop was tested at a given temperature, the upper and lower RPM bounds needed to be established by trial-and-error to ensure that the resulting torques met the above criterion. Eight more RPM values were chosen at roughly equal increments between the two bounds, so that a total of 10 measurements at various shear rates for each sample per test are projected. Three tests were conducted for every eye drop sample at each of the three temperatures. The average value of the measured viscosities at each shear rate under a specific temperature was used for analysis. Before a new eye drop sample was measured, the cup and spindle were both rinsed with distilled water and isopropyl alcohol and then air blow-dried.

The cone/plate rheometer induces a rheologically-controlled flow within the sample and measures its mechanical response to the shear deformation imposed by the rotating cone.^30^ More specifically, the cone is attached to a calibrated spring, which deflects as a result of the viscous drag of the eye drop solutions.^31^ As the cone spins with a given angular velocity, the velocity field within the sample approaches steady-state,^30^ and the angular velocity is converted to the desired rheological parameters. The angular speed of the rheometer is linearly correlated to the shear rate as shown below:

(1) $$\begin{array}{c} RPM=\frac{\dot{\gamma }}{7.5}. \end{array}$$

Five full rotations of the spindle were allowed before recording a measurement. For measuring the eye drops at ocular temperatures and 4.3°C, bath circulators were connected to and used with the rheometer (Fig. 1) to control the temperature of eye-drop samples.

## Results

**Figure 2.**
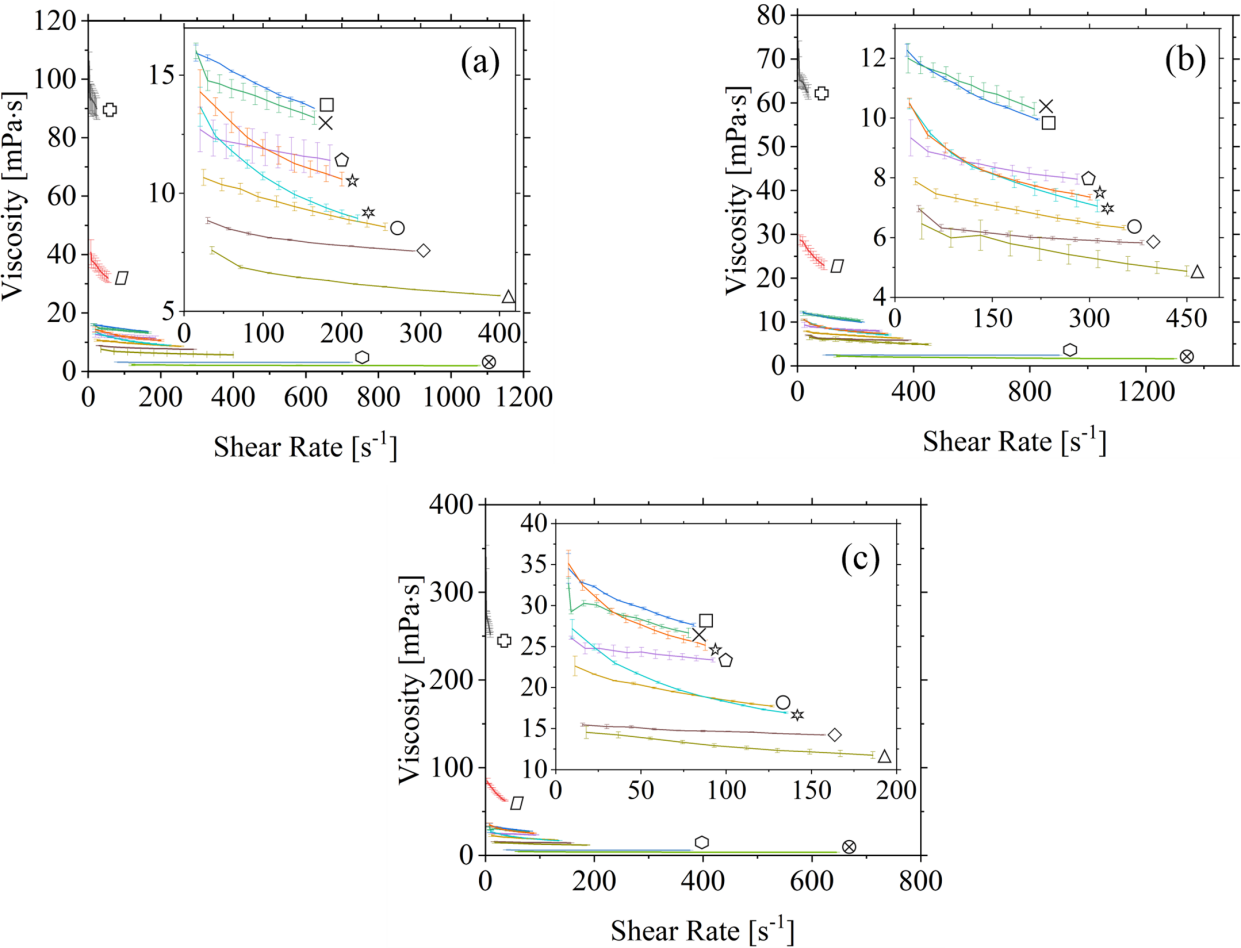
Shear viscosities of 12 different commercial eye drops at (a) 24.6 ± 1.2°C, (b) 34.5°C, and (c) 4.3 ± 0.3°C. Refresh Optive Gel Drops (*heavy cross*), Systane Ultra Hydration (*rhombus*), Refresh Optive Fusion (*square*), Blink Moisturizing Lubricant Eye drops (*X*), Bio True (*pentagon*), Long Lasting Relief (*circle*), Hylo Comod (*starburst*), Refresh Optive Advanced (*diamond*), Systane Complete (*triangle*), Systane Ultra High Performance (*star*), Thealoz Duo (*hexagon*), Systane Balance Lipid Layer Formula (*circled X*).

The shear viscosities of 12 commercially available eye drops were measured at three different temperatures within the minimum and maximum shear rates allowed by the rheometer, with three trials conducted at each temperature. Based on rheological analysis (Fig. 2), the shear-thinning behavior for all eye drops tested at each of the three temperatures was identified. Table 2 provides the average of the upper and lower bounds of the measured shear viscosities in the range of applied shear rates for the three relevant temperatures.

**Table 2.** Measured Shear Viscosity and Shear Rate Ranges for 12 Commercial Ocular Lubricants

	4.3°C		24.6°C		34.5°C	
Ocular Lubricants	Viscosity Range [mPa · s]	Shear Rate Range [s^−^^1^]	Viscosity Range [mPa · s]	Shear Rate Range [s^−^^1^]	Viscosity Range [mPa · s]	Shear Rate Range [s^−^^1^]
Refresh Optive Fusion	27.6–34.5	7.5–81	13.6–15.9	15–165	10.0–12.3	18.8–220
Long Lasting Relief	17.7–22.6	11.3–127	8.6–10.7	24.7–255	6.3–7.9	31.5–353
Refresh Optive Advanced	14.2–15.5	15.7–157	7.6–8.8	30–290	5.8–7.0	36.7–381
Systane Complete	11.8–14.5	18–186	5.7–7.6	35.3–400	4.9–6.5	41.3–450
Bio True	23.4–26.1	9–92.3	11.4–12.7	20.3–185	8.0–9.3	24.7–281
Systane Ultra High Performance	35.2–35.1	7.5–87.7	10.6–14.3	20.3–200	7.4–10.5	22.5–301
Thealoz Duo	5.8–6.2	38.3–376	3.1–3.2	80.2–720	2.4–2.5	97.5–901
Hylo Comod	16.9–27.2	9.75–135	8.9–13.7	20.3–220	7.1–10.5	22.5–312
Systane Ultra Hydration	62.2–84.8	3–36	31.9–40.8	6.75–54.7	22.9–28.7	9–91.5
Systane Balance Lipid Layer Formula	3.5–4.2	60–645	2.0–2.3	120–1072	1.6–2.2	135–1296
Refresh Optive Gel Drops	251.8–340.2	0.75–9	89.9–106.2	2.25–23.3	62.5–72.3	3.75–36.7
Blink Moisturizing Lubricant Eye drops	26.7–32.7	7.5–78	13.2–16.0	15–165	10.3–12.0	20.3–215

### The Effect of Temperature

In general, for the same sample at approximately the same shear rates, the measured shear viscosity increased with decreasing temperatures. At room temperature, Refresh Optive Gel Drops (Allergan Inc., Dublin, Ireland) had the highest viscosity (106.23 mPa · s) at the lowest measurable shear rate (2.25 s^−^^1^), and Systane Balance Lipid Layer Formula (Alcon Laboratories Inc., Geneva, Switzerland) had the lowest viscosity (2.25 mPa · s) over the lowest measurable shear rate (120 s^−^^1^).

There was a distinct temperature response in the measured shear viscosity and shear rates. In all samples, as the temperature decreased, the viscosity curve shifted upward, and the flow consistency index *K* increased. Furthermore, as the temperature decreased, the range of shear rates that the rheometer was able to render became narrow because of an increased torque for a given shear rate. It should be noted that the relative percentage change of the flow consistency index *K* (that is, the effective viscosity at 1 s^−^^1^) of each eye drop differed with changing temperature. To calculate this, first, the mean overlapping shear rate for a given eye drop sample at the three measured temperatures was determined. Then, the effective viscosity of each eye drop sample at the three temperatures at this shear rate was calculated using the Ostwald-de Waele fitted coefficients, so that a relative percent change could be determined. For example, when the temperature of Refresh Optive Gel Drops (Allergan Inc.) was increased from 4.3°C to 24.6°C, a 63% decrease in viscosity was observed at a shear rate of 5.63 s^−^^1^.

However, in the case of Systane Ultra Hydration (Alcon Laboratories Inc.) undergoing the same temperature change, a 49% decrease in viscosity was observed at a shear rate of 21.38 s^−^^1^. Similarly, when the temperature was increased from 24.6°C to 34.5°C, the viscosities of Refresh Optive Gel Drops and Systane Ultra Hydration decreased by 30% and 23%, respectively. In general, these differences can be attributed to the type and concentration of the constitutive macromolecules and the salinity of each eye drop formulation.

### The Shear-Thinning Property of Eye Drops and Mathematical Modeling

To quantify the shear-thinning behavior of the eye drops, the viscosity data can be modeled using the Ostwald-de Waele equation, which is derived from Newton's Law of Viscosity and has been generally used to describe the behavior of typical non-Newtonian fluids^32^

(2) $$\begin{array}{c} \mu_{eff}=K\left(T\right){\dot{\gamma }}^{n-1}, \end{array}$$

where *K* (equal to the viscosity at 1 s^−^^1^) is the flow consistency index (Pa s^n^) and is a function of temperature; and *n* is the dimensionless flow behavior index (the fluid is a pseudoplastic when *n* < 1).

**Figure 3.**
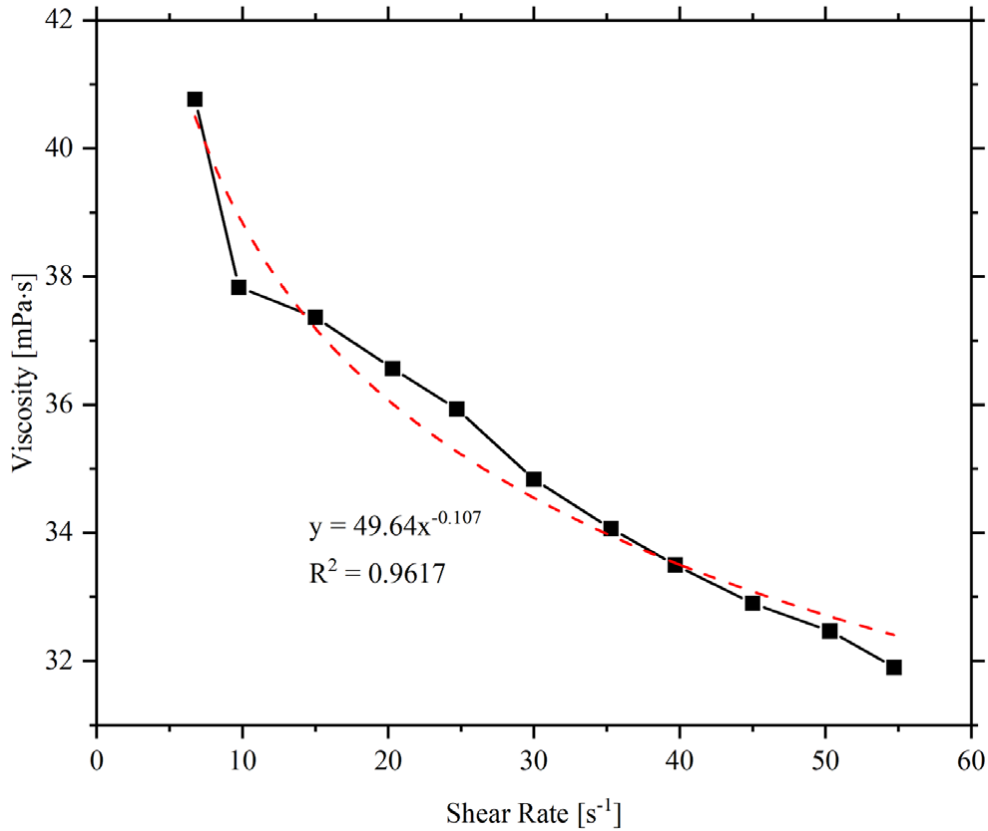
The power-law fitting (*dashed line*) of the shear viscosity data of Systane Ultra Hydration (*solid line*) at room temperature, where *K* and *n* are 49.64 mPa · s^0.893^ and 0.893, respectively.

To determine the coefficients (i.e., *K* and *n*) of the Ostwald-de Waele equation, a power-law formula was used to best-fit the viscosity data of every sample (Fig. 3). Based on the viscosity data for Systane Ultra Hydration (Alcon Laboratories Inc.), the flow consistency index *K* was found to be 49.64 mPa · s^0.893^, and the flow behavior index *n* was 0.893, indicating that this eye drop is a shear-thinning fluid. Similarly, all 12 eye drops at each of the three studied temperatures were analyzed, and the two corresponding characteristic indexes have been obtained. Table 3 lists the coefficients for each of the 12 samples at each of the three temperatures.

**Table 3.** Ostwald-de Waele coefficients for 12 commercial eye drops

	4.3°C			24.6°C			34.5°C		
Eye drops	*K*(T)	*n*	*R* ^2^	*K*(T)	*n*	*R* ^2^	*K*(T)	*n*	R^2^
Refresh Optive Fusion	42.22	0.91	0.95	19.77	0.93	0.90	16.09	0.92	0.95
Long Lasting Relief	30.37	0.89	0.98	15.71	0.89	0.93	10.99	0.91	0.96
Refresh Optive Advanced	17.70	0.96	0.93	11.47	0.93	0.99	8.58	0.93	0.93
Systane Complete	21.64	0.89	0.95	11.40	0.88	0.99	10.63	0.88	0.91
Bio True	28.75	0.95	0.99	14.73	0.95	0.97	11.65	0.93	0.98
Systane Ultra High Performance	48.95	0.85	0.99	23.71	0.85	0.98	16.35	0.86	0.99
Thealoz Duo	6.68	0.97	0.73	3.40	0.98	0.76	2.59	0.99	0.87
Hylo Comod	43.72	0.81	0.97	26.45	0.80	0.98	16.98	0.85	0.99
Systane Ultra Hydration	106.82	0.86	0.97	49.83	0.89	0.95	37.27	0.90	0.90
Systane Balance Lipid Layer Formula	5.74	0.92	0.99	3.04	0.94	0.98	5.07	0.84	0.98
Refresh Optive Gel Drops	290.23	0.94	0.74	109.99	0.93	0.86	74.24	0.95	0.77
Blink Moisturizing Lubricant Eye drops	33.79	0.95	0.63	19.49	0.93	0.97	15.54	0.93	0.91

It is noted that the coefficients of the Ostwald-de Waele relation are explicitly relevant across the range of shear rates under which the measurements were conducted. Therefore care must be taken when attempting to extrapolate the viscosity values at more physiologically relevant shear rates.

Other widely used mathematical models (like the cross and sisko models)^33^ could have been used to fit the viscosity evolution against shear rates, but these models require an infinite shear viscosity (η_∞_), which can only be found via plotting the data in a logarithmic manner (ie. by a log-log plot) and locating the second Newtonian plateau.^34^ However, because of limitations in the range of shear rates rendered by the rheometer, this value was unable to be obtained. Thus a power-law model was chosen and used to fit all the data. The *R*^2^ value, which is a quantitative correlation coefficient between the model and the measured data, indicates that the power-law model has accurately described the relation of the shear viscosity and the shear rate.

## Discussion

It has been argued that eye drops having long residence times on the ocular surface provide the maximum benefit to the patient.^35^ Furthermore, it has been shown that at low shear rates, such as when the eye is open, patients can benefit from higher-viscosity artificial tears because of their greater residence time.^33^ Eye drops that have low viscosities at low shear rates may evaporate more readily and are susceptible to rapid drainage. Conversely, at higher shear rates, such as during blinking, a reduced viscosity of the eye drops is required to improve the comfort for the wearer and to decrease friction-related inflammation.^36^

### Viscosity-Shear Rates: Complexity and Irreversibility

Generally, artificial tears are non-Newtonian, shear thinning (pseudo-plastic) fluids, in which the viscosities decrease with the increased velocity of the eyelid movements. All the commercial eye drops tested in this study exhibited this shear-thinning behavior. On the low end, an eye tremor produces shear rates of 0.03 to 0.14 s^−1^, and on the high end, a blink can result in estimated shear rates of 4250 to 28500 s^−1^.^37^

An eye drop's low-shear viscosity is a result of the stabilization promoted by polymer chain entanglement. Under high-shear conditions, chain disentanglement in the direction of shear occurs, leading to fewer molecular interactions and an overall reduced viscosity.^26^ At a critical shear rate or shear stress, there is a distinct drop in viscosity, which indicates the beginning of the shear-thinning region. At low shear rates, the macromolecules in artificial tears maintain their irregular order because of molecular interactions and Brownian motion. At high shear rates, the macromolecules stretch and align with the flow, causing a decrease in intermolecular interactions, which contributes to a drop in viscosity.^38^

Many artificial tear formulations contain the same lubricant macromolecules to improve their viscosities. For example, Long Lasting Relief (Hydrasense, Mississauga, Ontario, Canada), Hylo Comod (Hylo Eye Care, Saarbrücken, Germany), and Bio True (Bausch + Lomb, Rochester, NY, USA) all contain sodium hyaluronate. However, these formulations are rheologically different from one another because of differences in their mass average molecular weight, polydispersity, and interactions between the lubricant and the other ingredients in the artificial tear.^34^

Shear thinning is normally reversible in most polymer solutions, and they will be able to regain their initial viscosity when the shearing force is eliminated.^32^ In some cases, the microstructure of the solution does not rebuild instantaneously after a reduction in the applied shear rate. In the case of artificial tears, the viscosity must be sufficiently low during the peak acceleration of the blink but then must quickly recover its viscosity at the end of the blink cycle. In this study, the shear viscosity was measured from the lowest to the highest shear rate in a stepwise manner. However, when the shear rate was quickly reduced from the highest back to the lowest starting shear rate, a slightly higher viscosity than had been previously measured was observed. This hysteresis phenomena demonstrates that eye drops, unlike single-component polymer solutions, are characterized with certain irreversibility in the shear-thinning property.

### Viscosity-Temperature: Significance to Applications

The shear viscosities of common lubricants used in eye drop formulations has been shown to vary with temperature by differing amounts.^39^ For example, in a study by Rahman et al.,^39^ it was shown that the decrease in the relative viscosity of 0.4% sodium hyaluronate was appreciably more than 0.5% carmellose sodium when the temperature was increased from 22°C to 35°C. Higher temperatures may induce greater mobility of the constituent macromolecules, leading to a decrease in the overall viscosity of the eye drop.

The blur threshold for artificial tears is believed to be approximately between 20 to 30 mPa · s.^34^ Above this threshold, eye drop users may potentially experience reduced vision, stickiness and a build-up of residue.^40^ Of all the artificial tears tested at room temperature, only Systane Ultra Hydration (Alcon Laboratories Inc.) and Refresh Optive Gel Drops (Allergan Inc.) exceeded the upper threshold. Examination of Table 2 would also suggest that when Refresh Optive Fusion, Systane Ultra High Performance, Hylo Comod, Systane Ultra Hydration, Refresh Optive Gel Drops, and Blink Moisturizing Lubricant Eye drops are applied directly after refrigeration, there could be a brief period of blurring. However, it is anticipated that this will quickly vanish once their temperatures increase on contact with the corneal surface.

### Limitations of the Current Study

This study only measured the effects of temperature, shear rate, and viscosity of 12 eye drops. Further studies may need to include a wider range of available artificial tears (with and without preservatives) and look at other physiochemical properties like osmolarity, surface tension, density and molecular weight.^24^ Furthermore, the normal stress difference (N1) with increasing shear rate should be measured to determine the effects of the blink cycle on artificial tears.^33^ The technical limitations of this study are that the rheometer used could not measure the viscosity of the samples within a consistent shear rate range, nor could it reach shear rates all the way up to 28,500 s^−1^. For this reason, caution must be taken when extrapolating the results of this study beyond the measured shear rates. Furthermore, it is important to note that the in vivo shear behavior of these ocular lubricants has not been investigated. A separate study related to this would be beneficial to get a more comprehensive view of their rheological behaviors at a range of physiologically relevant shear rates and temperatures. This is important because it is believed that the minimum high-shear viscosity needed to maintain a corneal coverage is 10 mPa · s.^34^

### Clinical Relevance

To mitigate the symptoms of DED, it is believed that as a starting point, practitioners should select an eye drop that has shear-thinning non-Newtonian behaviour that is a high viscosity at low shear rates and a significant percentage drop in viscosity at high shear rates.^36^ An eye drop with this property would theoretically be ideal because it would satisfy the requirement for efficacy (providing sustained hydration) while providing the user with maximum comfort and vision. From the 12 eye drops studied, Refresh Optive Gel Drops (Allergan Inc.) had the highest overall room temperature viscosity, as well as the greatest percentage drop in viscosity (30%) from room temperature to ocular surface temperature (34.5°C). Of the eye drops with a room temperature viscosity below the blur threshold, both Bio True (Bausch + Lomb) and Systane Ultra High Performance (Alcon Laboratories Inc.) had the largest percentage drop in viscosity (27% and 28%) between room temperature and ocular surface temperature. Furthermore, the average percentage decrease in the viscosity from refrigeration to ocular surface temperature was 59%. The two eye drops with the largest drop in viscosity from 4.3°C to 34.5°C were Refresh Optive Gel Drops (Allergan Inc.) (74%) and Systane Ultra High Performance (Alcon Laboratories Inc.) (65%). However, it should be noted that because shear viscosity is clearly not the only factor related to eye drop comfort and performance, clinical studies related to the qualitative assessment of drop comfort and overall performance are required to verify this guidance in practice.

## Conclusion

The shear viscosity of an artificial tear is a key property that dictates residence time and may be linked to both user comfort and vision quality, which could impact patient compliance and acceptance.^24^ In this study, the rheological behavior of 12 artificial tears at three clinically relevant temperatures was studied, and a power-law model was developed for each formulation. The formulations varied in their chemical composition and, as such, were featured with significant differences in their measured shear viscosities. All samples exhibited the behavior of being shear-thinning, (i.e., viscosity tended to become smaller as the shear rate increased), an ideal feature for an artificial tear. This behavior of each eye drop sample was further closely modelled by the Ostwald-de Waele equation ($\mu_{eff}=K\left(T\right){\dot{\gamma }}^{n-1}$), with two characteristic indexes, namely, the flow consistency index *K* and the flow behavior index *n*. The specified models might allow the estimation of viscosities of the commercial eye drops at even higher shear rates, (e.g., those that are physiologically relevant but are generally difficult to achieve experimentally).

Refrigerated storage of artificial tears before use is frequently undertaken by patients to provide a cooling sensation on insertion on the ocular surface in an attempt to relieve discomfort and reduce inflammation caused by the onset of DED. However, this results in a marked departure in shear viscosity from that seen at room temperature and may not be an ideal practice to undertake. When the artificial tear is applied to the corneal surface, its temperature will increase rapidly, causing a significant change in its viscosity. The results indicate that there is an inverse relationship between temperature and viscosity of the eye drop. Of the 12 eye drops studied, there was a maximum decrease of 63% when the temperature was increased from 4.3°C to 24.6°C (shown by Refresh Optive Gel Drops), and a maximum decrease of 30% from 24.6°C to 34.5°C (shown by Refresh Optive Gel Drops). Our results have quantified the degree to which the shear viscosity changes with these relevant temperatures, which may help clinicians decide on the potential for temperature-mediated viscosity to impact the clinical effectiveness of the eye drop products they prescribe.
